# EXPRESSION OF CONCERN: Apoptotic Efficacy and Antiproliferative Potential of Silver Nanoparticles Synthesised From Aqueous Extract of Sumac (*Rhus coriaria* L.)

**DOI:** 10.1049/nbt2/9856498

**Published:** 2026-06-28

EXPRESSION OF CONCERN: P. Ghorbani, F. Namvar, M. Homayouni‐Tabrizi, et al., “Apoptotic Efficacy and Antiproliferative Potential of Silver Nanoparticles Synthesised From Aqueous Extract of Sumac (*Rhus coriaria* L.),” IET Nanobiotechnology, 12, (2018): 600–603. https://doi.org/10.1049/iet-nbt.2017.0080.

This Expression of Concern is for the above article, published online on 27 March 2018 in Wiley Online Library (wileyonlinelibrary.com) and has been issued by agreement between the journal Editor‐in‐Chief, Duo Lin; and John Wiley & Sons Ltd. The Expression of Concern has been agreed due to an error identified in Figure 2b, in which unexpected, repeated elements are shared between the 5 and 15 µM panels [1].

During an investigation by the publisher, the authors were unable to provide the raw data or original images. The authors insisted that the duplication was a result of an error in image preparation, and it was determined that the error did not significantly affect the conclusions of the study. Therefore, the journal has decided to issue an Expression of Concern to inform and alert readers.

The authors have been informed about this Expression of Concern.
